# Using clinical simulation to study how to improve quality and safety in healthcare

**DOI:** 10.1136/bmjstel-2018-000370

**Published:** 2018-09-29

**Authors:** Guillaume Lamé, Mary Dixon-Woods

**Affiliations:** THIS Institute (The Healthcare Improvement Studies Institute), University of Cambridge, Cambridge, UK

**Keywords:** simulation-based research, research methods, healthcare improvement research, clinical simulation, quality of care

## Abstract

Simulation can offer researchers access to events that can otherwise not be directly observed, and in a safe and controlled environment. How to use simulation for the study of how to improve the quality and safety of healthcare remains underexplored, however. We offer an overview of simulation-based research (SBR) in this context. Building on theory and examples, we show how SBR can be deployed and which study designs it may support. We discuss the challenges of simulation for healthcare improvement research and how they can be tackled. We conclude that using simulation in the study of healthcare improvement is a promising approach that could usefully complement established research methods.

## Introduction

Simulation in healthcare can be broadly defined as a ‘tool, device, and/or environment (that) mimics an aspect of clinical care’.^1^ It has a long history in healthcare education,^1^ valued for its ability to reproduce some of the conditions of clinical practice and enable learners to practice in a safe environment. More recently, it has been used as a technique for supporting improvement in healthcare systems and processes, for example, by helping to diagnose problems or test new approaches before they are deployed for real.^2 3^ Given this history, most research is *about* simulation (focusing, eg, on its effectiveness in achieving training or practice goals) rather than *through* simulation.^4^ Despite some recent encouraging examples,^5 6^ the potential of simulation for *conducting* research has remained underexploited. In this article, we identify simulation-based research (SBR) as a distinct research strategy that seeks to generate scientific knowledge about human and organisational behaviour through use of simulation techniques that may take diverse forms, and we explore the role of SBR in the study of how to improve quality and safety in healthcare^7–11^ by offering an informal overview of relevant literature.

We build on the methodological literature in healthcare improvement research to frame our discussion of research designs and strategies, and we draw from the literature on simulation-based training in clinical settings to present the practical side of designing and delivering simulations. As our focus is on simulation for the conduct of research, we do not consider the now large literature on simulation as a training/education method or as a method of undertaking quality improvement. Articles included in the review were selected on the perceived insights they could generate for improvement research, using the expertise of the authors.^12^

## Simulation-based research designs

Different research designs are made possible using simulation-based techniques.^13 14^ We start by offering a broad overview of how simulation might be used in research, and then provide a short description and examples of three types of studies (descriptive, theory-testing and generation, and evaluating interventions) that might deploy simulation. We discuss the particular issues that may apply in multicentre studies. Finally, we discuss the potential for combining SBR with other methods in mixed-methods studies.

### Simulation as a way of studying clinical settings

Simulation for research classically seeks to reproduce features of a real-world phenomenon so that it can be studied. In researching how to improve quality and safety in healthcare, an important strategy involves the use of structured scenarios that set up specific settings or events that evoke or replicate features of real-world clinical situations,^1 15^ with the aim of producing data that can be analysed for purposes of generating applied or theoretical knowledge, or both.^9^

A simulator is the *medium* through which a simulation scenario is delivered to participants.^16 17^ Simulators are of many different kinds, including (but not limited to) manikins (which may be more or less interactive), study cases and actors playing the role of patients (so-called ‘simulated patients’).^18^ The choice of simulator depends on the research goals and on the clinical situation under study. Generally, the aim is that participants’ reactions and behaviours be as close as possible to what they would experience and do in a real situation, so their perceptions of events, timing, environment and cues are all important.^16 19^

Simulations may take place either in dedicated simulation centres or in situ, in real-life clinical settings. Recommendations for the design of simulation centres suggest taking a range of factors into consideration, including layout of the different spaces, the design of the control room or noise insulation.^20^ In situ simulations, which take place in people’s own workplaces, have a number of strengths; for example, one study was able to identify latent safety threats in an emergency department using unannounced simulations that were organised during normal shifts.^21^ However, in situ simulation is not free of challenges: it may, for example, interfere with care or disturb patients and staff.^21 22^

Various types of data can be collected during a simulation that may be used for research purposes. Simulators themselves often collect data: high-fidelity manikins, for example, can record data such as compression or body position, while laparoscopic simulators can use movement-tracking to assess dexterity.^23^ Physiological measurements of participants’ reactions may include monitoring of skin resistance as an indicator of the activity of the sympathetic nervous system^24^ or assessing the cortisol level of participants to measure stress.^25^ Researchers may also use observational checklists to code the behaviour of simulation participants (eg, box 1).^26^ Pre-post questionnaires are widely deployed: in a study of the impact of stress on the performance of paramedics, for example, LeBlanc *et al* used questionnaires to measure anxiety before and after simulation.^25^

Box 1 **Randomised trial on the impact of training on speaking-up behaviour**

- Although individuals speaking up about concerns is often the final barrier to adverse events in most high-risk industries, previous research has shown that many hurdles prevent individuals from giving voice healthcare. Raemer *et al* 88 investigated the impact of a 50 min training workshop on the ability of anaesthesiologists to speak up in the operating room. The intervention group had the ‘speaking-up’ training before the simulation; for the control group, it was after the simulation. The scenario included three events where anaesthesiologists had the opportunity to speak up to a surgeon, a nurse and another anaesthesiologist, respectively. Simulations were video-recorded, and the reactions of participants to the three events were coded based on the videos. The debriefing sessions were also videotaped, and the hurdles and enablers to speaking up mentioned by participants and passive observers of the simulations were coded based on an existing taxonomy.
- There was no significant difference between the control group and the experimental group on any of the reactions to the three events. The main hurdle to speaking up was ‘uncertainty about the issue’—a surprise for the authors because participants had on average 15 years of experience. In conclusion, the authors acknowledge that the intervention was not effective, and recommend more education on, and institutional support for, speaking up. More generally, these results challenge the efficacy of relying solely on educational interventions to improve speaking-up behaviours.

Qualitative data from simulations can also be collected either in real time or through use of video recordings. The think-aloud technique, whereby participants verbalise their thoughts during the simulation, is an example of one commonly used method.^27^ Post-simulation debriefing sessions are also valuable sources of data, as illustrated in box 1. Taking place after a simulation has ended, debriefs may involve feedback to the participants, who are in turn invited to discuss their experiences.^28 29^ One-to-one interviews or focus groups may also be used.^8^

### Descriptive studies

One important role for SBR is in understanding what happens in healthcare organisations and why, perhaps by reproducing a situation or condition of interest in a simulation. Researchers may design a simulation to reproduce current practice in a controlled environment in order to observe specific aspects. The aim is to document individual and collective behaviour, and to identify patterns of interactions and thought processes. For instance, Fossum *et al* 30 used written patient cases as a simulator to investigate nurses’ thinking strategies when faced with malnutrition and pressure ulcers in nursing homes. Box 2 describes a study on information sharing in surgical teams.

Box 2 **Exploratory study on information sharing in surgical teams**

- Collaboration and teamwork are essential elements of safe care. Cumin *et al* 89 investigated the sharing of information between individuals in a surgery team. Twenty teams of six people participated in surgical simulation. Before each simulation, participants received an individual case briefing note. Each team member had in their briefing note a specific piece of information that the others did not have, but which was clinically useful for managing the simulated case. Researchers counted if, and when, the information items were shared with the rest of the team. They also tested team members about the information items after the simulation, using a questionnaire.
- Team members were 5.0 times (95% CI 1.5 to 18.4, p=0.01) more likely to remember an information after the simulation if it had been shared during a formal communication time rather than at another time. However, in a significant number of instances information items were not shared (38%), and not all members participated equally (anaesthesiologists and senior surgeons were more likely to share information). The results support the importance of formal communication slots before surgery, but still highlight insufficient information sharing and imbalance between team members.

The output can be a *descriptive account*, as, for instance, in a study of how professionals share information in the operating room (box 2), or how nurses make decisions about deteriorating patients.^31^ Used in this way, a distinctive strength of SBR is that it can generate data that could not be obtained with other methods, enabling study of issues that are not possible using other methods for ethical, practical or safety reasons.^7^ It may be especially useful in the study of rare events, where it is not feasible for researchers to wait for a rare event to occur and hope to be there to observe it—and observe it only once. In a simulated environment, rare but potentially catastrophic events or conditions can be reproduced as often as desired, potentially under varying conditions. For instance, simulation has been used to observe how care teams managed malignant hyperthermia, a rare life-threatening condition.^32^

### Theory-testing and generation

Simulation-based studies can be useful when the aim is to generate, evaluate and extend theories relating to quality and safety. For example, one important hypothesis based on studies in cognition and human biology is that sleep deprivation has adverse effects on the performance of physicians.^33^ SBR has made a useful contribution to investigating this hypothesis (box 3). The impact of noise on anaesthetists’ stress level in operating theatres has also been studied using simulation.^34^ Similarly, social and cognitive psychology studies have generated hypotheses about the influences of peer pressure, including the possibility that individuals will conform to a group’s opinion even if they think it is false. Simulated experiments with healthcare students have shown how this could affect clinical practice.^35 36^

Box 3 **Randomised trial on the impact of sleep deprivation on non-technical skills**

- The impact of sleep deprivation on the performance of anaesthetists is still uncertain, and research so far has mainly focused on technical skills. Neuschwander *et al* 79 studied the impact of sleep deprivation on non-technical skills, including ‘team working, situation awareness, decision-making, and task management’. The authors developed a crisis management simulation scenario, using a high-fidelity manikin. Also, 10 participants undertook the scenario after a night shift and 10 after a rested night. Two blinded assessors rated the performance of participants using a validated scoring tool.
- The non-technical skills score was significantly lower for the sleep-deprived anaesthesiologists. In particular, team working scores were significantly lower. Self-confidence in anaesthesia skills just before the simulation was also significantly lower in the sleep-deprived group. These findings are important since non-technical skills are suspected to play a key role in avoiding serious adverse events. This study also illustrates the difficulty of recruiting when participation is voluntary: 100 participants were screened, but only 21 agreed to participate. However, the authors argue that the significant difference in non-technical skills makes lack of power unlikely.

### Evaluating interventions

A particularly attractive role for simulation is in evaluating interventions that seek to improve care, not least by affording experimental and other study designs that would otherwise be difficult or impossible to do in real life. Table 1 identifies possible deployments of simulation in the different study types identified by Portela *et al*.^13^ Some SBR studies use pre-post designs, usually involving an initial observation of the phenomenon of interest performed in a simulated environment, followed by the introduction of an intervention (training, new procedure, new equipment, etc) and repeated measures taken in the same simulated environment (c.f. box 4).

**Table 1:** Roles for simulating in different study types aimed at evaluating improvement interventions

Class of studies	Potential role(s) for simulation	Potential data collection method(s)	Example(s)
Quality improvement projects	Understand the problem situation Assess intervention feasibility Evaluate the effect of the intervention Optimise design and implementation of the intervention	Observational checklists Focus groups and interviews Simulator-collected measures Physiological measures Questionnaires	Combination of simulation and Failure Modes and Effects Analysis in a prospective risk analysis^90^ Identification of latent threats in a new hospital facility^91^
Effectiveness studies: *Randomised controlled trials (RCTs)* *Quasi-experimental studies* *Observational studies*	Evaluate the effectiveness of the intervention Pre-test interventions	Observational checklists Simulator-collected measures Physiological measures Questionnaires	*RCT:* simulation-based trials of surgical checklists,^6 46^ trial on the impact of sleep deprivation on non-technical skills (box 2),^79^ trial on the impact of training on speaking-up behaviour (box 1)^88^ *Quasi-experimental studies*: uncontrolled study of a new drug packaging system (box 4),^92^ uncontrolled evaluation of a paediatric resuscitation training package,^93^ non-randomised controlled study of an intervention to improve the management of distractions and interruptions during ward rounds^94^ *Observational studies:* simulation-based longitudinal study of crisis resource management ability^95^
Process evaluations	Evaluate how the intervention is received by participants	Observational checklists Focus groups and interviews Simulator-collected measures Physiological measures Questionnaires	Use of simulated patients as part of a multimethod process evaluation of an intervention to improve youth‐friendly services for sexually transmitted infections^96^
Qualitative studies	Explore perceptions of the intervention Produce descriptions and theoretically informed analysis of scenarios	Focus groups and interviews (Video-)ethnography	Qualitative study of the simulation of an audio-visual telehealth service^97^
Economic evaluations	Feed data to economic models and projections	Observational checklists Simulator-collected measures Physiological measures Questionnaires	Use of clinical simulation in the cost-effectiveness evaluation of an electronic health record system^98^

Box 4 **Uncontrolled before–after study of a new drug packaging system**

- Medication errors are a leading cause of adverse events in hospitals. Garcia *et al* 92 studied the impact of a new labelling system using a simulated medicine room. For 30 min, each participant was handed a new medication chart once he/she had completed preparation for the previous one. Researchers timed the preparation of each medication chart using a stopwatch and counted the number of errors in preparation with the standard labelling system. They repeated the experience 3 months later, using a new labelling system proposed in the literature by Endestad *et al*.^99^
- The error rate remained low with no significant change, but nurses were significantly quicker in their preparation with the new labelling system. These results contrast with a previous on-screen experiment, where the error rate deceased with the new system.^99^

The ability of SBR to reproduce situations identically before and after increases confidence that the intervention can explain the variation in outcomes of interest. However, in general, simple uncontrolled pre-post studies suffer from weaknesses in attributing causality to the intervention.^14 37^ Time-series designs, where multiple data points are collected before and after the intervention,^14^ are generally preferred, but even better, when the aim is to attribute causality, are controlled designs.^13^

Simulation is especially well-suited to facilitating controlled studies, which expose one group but not the other(s) to the intervention(s) (boxes 1 and 3). Controlled studies using simulation have been used, for example, to compare semiautomated defibrillators and automated external defibrillators for the management of in-hospital sudden cardiac arrest.^38^ Simulation may also have some role in supporting process evaluations, which look at how the intervention is implemented and received.^39 40^

### Multicentre studies

Cheng *et al* provide a guide for multicentre simulation studies,^41^ which offer several advantages. The ability to standardise the simulation across participating sites helps to isolate independent variables and to reduce the risk of bias introduced by variations in local contexts.^41^ Because multicentre studies provide larger sample sizes, they can increase the generalisability of findings, and enable comparisons between sites.^41^ For instance, one study investigated variability in chest compression during paediatric cardiopulmonary resuscitation (CPR) in nine hospitals.^42^ It found that the quality of CPR varied between hospitals even when a just-in-time training intervention was delivered, thus calling into question the effectiveness of current guideline implementation strategies and training approaches for improving discrepancies in care quality. Box 5 shows an example of a multicentre simulation-based study.

Box 5 **Multicentre, cross-sectional observational study of paediatric sepsis management**

- Severe sepsis is a major source of morbidity and mortality in paediatric patients. Adherence to associated guidelines has been shown to improve outcomes. Kessler *et al* 100 compared the practice of 47 teams in 24 emergency departments (EDs) on the management of paediatric sepsis. The teams came from both paediatric and general EDs. Simulations were conducted in situ, and the primary outcome was adherence to a guideline measured by a six-component checklist. The investigators’ hypothesis was that paediatric EDs would adhere to the guideline more than general EDs.
- Paediatric EDs demonstrated greater adherence to the guideline than general EDs. Nonetheless, in an adjusted regression analysis the only factor associated with greater adherence was composite team experience (mean number of years of experience as a medical professional for each team), suggesting that hospitals should give priority to this factor when trying to improve paediatric sepsis management.

### Mixed-method strategies

Simulation can be used both as a stand-alone method or as a complement to other research strategies.^13^ In healthcare improvement research, multimethod approaches are important when studying ‘dynamic, complex and interacting systems in which innovations in [Quality Improvement] are implemented’.^43^ For instance, a problematic situation might be analysed through ethnography, structured observation methods and hazard analysis approaches to identify and characterise the problems and opportunities for intervention; simulation may then be used to evaluate a candidate intervention in a pilot study. If the simulation confirms that the intervention impacted outcomes, these findings would be generalised qualitatively: the causal relationship found in the simulation would be generalised, but not the quantitative strength measured during the simulation.^44^ Finally, a multicentre controlled study could increase external validity. Process evaluation can be used to assess what happened in practice, fidelity to intervention design and implementation challenges.^13^ This kind of combination of methods provides a robust framework for evaluation, allowing triangulation and mitigating the weaknesses of each independent method.^45^ For instance, the impact of surgical safety checklists on care quality has been explored both through simulation,^6 46^ clinical trials^47 48^ and qualitative research.^49^

## Considerations in simulation-based research

Simulation as a research method has some distinctive advantages. It also poses a number of challenges: practical, pertaining to validity and fidelity, reporting and ethics.

### Practicality

The practical organisation of SBR can be challenging. One barrier is the potential cost.^7^ High-fidelity manikins and simulation facilities come at an often high price. Lower-fidelity simulators are less expensive, but may not offer the same possibilities (physical and functional resemblance to real patients, real-time measurements of the manikin’s state). Simulation also requires space and facilities, while setting up and managing the simulator may involve specific expertise.^50^ Once the study has been designed, recruitment can also be an issue, especially without leadership support.^7^ The lack of faculty protected time can be a barrier towards participating in or organising simulations.^51^

### Methodological challenges: validity

SBR poses specific challenges concerning validity compared with more traditional research approaches. *Internal validity* refers to the ability to show that correlations between observations in a study are causal in nature.^52^ In other disciplines like psychology, economy or management research, the strength of behavioural laboratory experiments is often thought to lie in their internal validity.^52–55^ For SBR to have high internal validity, the design and reporting of studies needs to be handled carefully.^1 56 57^ In particular, scenarios need to be carefully described so that simulations are as standardised as possible.^7^ For instance, in the study of a new drug packaging system described in box 4, it was important that the simulations performed before and after the introduction of the new packaging differed as little as possible, so that changes in performance could be attributed to the new packaging system rather than to other elements. If the presentation of the mediation charts had been modified, or if the organisation of the drug storage system had been changed, these changes may have affected performance. This would have made it difficult to conclude on the impact of the labelling system.

To ensure consistency in replications of the same scenario, frameworks used to describe scenarios in simulation-based education may, with some adaptations, be suitable for clinical simulation research. For instance, the TEACH Sim framework covers simulation objectives, audience, patient details, simulator details, scenario script (sequence of events and the expected reaction of the learner), equipment, confederates and actors, and team composition.^58^ When the simulation involves confederates or actors (eg, in the roles of patients or other healthcare professionals), they must be trained in order to reproduce the same behaviour in each simulation, and to respect the scenario script.^7^ To ensure that they unfold as intended, scenarios should ideally be pilot-tested.^59^

*External validity* (applicability or generalisability)^60 61^ describes ‘whether causal relationships can be generalized to different measures, persons, settings, and times’.^62^ Clearly, the generalisability of research findings generated in a simulated environment to ‘real life’ and patient outcomes is an important question.^7 9^ It is of particular concern in SBR, because, like laboratory experiments,^52–55^ SBR artificially reduces the environmental complexity surrounding the studied phenomenon, potentially weakening confidence in how far it is possible to generalise from these highly controlled conditions.^63 64^ For instance, although some studies specifically address the issue of interruptions in clinical practice,^65^ other studies will attenuate this dimension to put the emphasis on other aspects, such as clinical complications. However, in everyday practice, both factors interact: complications happen and interruptions occur, sometimes simultaneously and other factors such as social relationships also play a role. It is important to know to what extent the results of the study on interruptions would hold in this much richer context.

One way of evaluating the external validity of lab experiments is to compare them with related field studies.^53 66^ In psychological research, the findings suggest that laboratory experiments are best suited to establishing the existence and direction of effects between variables, not the precise magnitude of the effects measured in the lab.^53 66^ This cautious approach has also been recommended in economics.^44^

An alternative view of external validity is that it should depend on the research aims and, since ‘real life’ is not neutral, objective phenomenon, field observations should always be understood as socially constructed and theoretically informed.^54 63^ Finally, when critiques argue that lab experiments create a strong observer effect (a modification of the behaviour created by the presence of an observer, sometimes called the ‘Hawthorne effect’), promoters of lab experiments respond that field research also involves observers, whose presence influences behaviour.^63^ Therefore, the difference between lab experiments and fieldwork is less clear-cut than it is sometimes presented.

*Construct validity* is ‘the degree to which a score can be interpreted as representing the intended underlying construct’.^67^ Construct validity is not a binary notion; it is built with accumulating evidence to support interpretations of assessment data.^68^ SBR does not seem different from other improvement research approaches regarding construct validity, although Cook highlights the need for validity studies on measurement tools used in simulation (eg, scoring tools or checklists).^26^

### Methodological issues: fidelity

*Fidelity* broadly refers to the extent to which a simulation reproduces the experience of the real-world situation it aims to replicate. The concept is problematic, often ill-defined and used in a binary way (‘high’ or ‘low’ fidelity),^16 19^ whereas a closer analysis shows that the notion is more complex. Certain aspects of the ‘real world’ may be more important in some studies, allowing more freedom and less resemblance on other dimensions.

Beaubien and Baker describe fidelity on three dimensions: psychological, environment and equipment.^69^ Tun *et al* propose to decompose fidelity into three dimensions: patient fidelity (the extent to which the simulator mimics actual patient behaviour), clinical scenario fidelity and healthcare facilities fidelity. Rather than focusing only on high-technology manikins with elaborate physiological responses, these authors insist that all three dimensions of fidelity are important in creating ‘an accurate representation of real-world cues and stimuli’.^16^ Hamstra *et al* insist on *functional task alignment* as a necessary complement to *physical resemblance*.^19^ But even physical resemblance can be multifactorial. For instance, very simple and low-cost surgical simulators can be useful, as in the example of chicken breast used to teach ultrasound-guided vascular access.^70^ Chicken breasts do not even remotely look or feel like human bodies, but their physical properties replicate the effect of ultrasound better than high-fidelity manikins.^70^ This can make chicken breasts a more appropriate simulator when the focus is on the ultrasound dimension: they allow for the right actions to be performed (*functional task alignment*) and adequately replicate the physical parameters of interest (*physical resemblance*).^19^

As in the use of simulation for education,^16 19 69^ important considerations for fidelity in SBR include the perceived realism/authenticity of the scenario, the patient/simulator and the simulation environment.^7 16^ Researchers need to ensure that the simulation is sufficiently authentic (in terms of physical resemblance and functional similarity)^19^ to enable comparisons with participants’ ‘real-life’ behaviour. They should also pay attention to the *phenomenal* (emotions, beliefs) and *semantical* (meaning, theories, information) content of scenarios, before focusing on the *physical* dimension.^71^ All three aspects are important and will affect the behaviour of participants.

Though it is sometimes proposed that high-technology manikins might lead to better learning outcomes than less elaborate simulators,^19^ evidence of the relationship between the physical fidelity of the simulator and the learning outcomes in simulation-based education is thin.^72^ The choices largely depend on the situation to be studied and the objectives of the simulation. For instance, when studying surgical dexterity, it may be important to reproduce human anatomy, and high-fidelity manikins, cadavers or virtual reality will be appropriate.^23 73^ Similarly, when training for highly technical skills in obstetric emergencies, ‘high-fidelity’ manikins generate better results than simpler ‘doll-like’ manikins.^74^ However, video vignettes, an apparently less sophisticated medium, proved appropriate when studying general practitioners' decisions to investigate suspected lung cancer,^75^ because they provided enough relevant information and resemblance with the real task. The choice of the setting for the simulation can also be important, for instance, by choosing to organise simulations in situ, that is, where care is routinely performed,^22^ rather than in a simulation lab.

Researchers should of course be aware that the behaviour of participants in a simulation may not reproduce what they would do ‘in real life’. For instance, the level of psychological safety experienced by participants may affect their engagement with the simulation.^76 77^ The style of debriefing may also affect the outcomes, in particular if judgement is involved.^29^

### Reporting

Recent literature reviews have highlighted the shortcomings of quantitative studies on simulation-based educational interventions, in terms of both reporting (simulation context, outcomes, statistical methods) and statistical analysis.^1 56^ This suggests a need for attention to quality of reporting for SBR. Researchers should be aware of extensions to the Consolidated Standards of Reporting Trials statement for randomised trials and the Strengthening the Reporting of Observational Studies in Epidemiology statement for observational studies.^78^ These guidelines recommend that researchers should specify whether they are reporting *research on simulation*, or use *simulation as an investigative method*. Further, the guidelines suggest that generalisability of the findings from simulation outcomes to patient outcomes should be explicitly discussed.

### Ethics

A particular strength of SBR is that it enables studies of quality and safety without putting real patients (or staff) at risk. For instance, it might not be acceptable to assess the effects of sleep deprivation on a physician’s performance directly with real patients, but SBR has been used to evaluate the link between sleep duration and the skills of surgeons or anaesthesiologists (box 3).^23 79^ New procedures and systems can be evaluated in a safe environment without threatening patient care, for example, allowing identification that a new drug labelling system could have generated more medication errors (box 4). Similarly, scenarios can be allowed to unfold, even when a researcher has noticed a dangerous situation developing—something that would be unacceptable in direct observation (box 1).

SBR is not free of ethical consequence, however. For instance, during in situ simulation, the person recording should avoid filming bystanders, as this raises privacy issues.^22^ Perhaps less straightforward are issues relating to adverse outcomes and the use of deception.^80–83^ Calhoun *et al* report a simulation scenario combining both issues, where a confederate acting as a senior clinician ordered the administration of the wrong medication for the simulated case.^81^ The objective was that the participants would challenge this instruction even though it meant overcoming hierarchical distance. If they went on to administer the wrong drug, the simulated patient died. This kind of deception can be problematic because participants are asked to ‘suspend disbelief’ during simulation, but are then confronted with potentially traumatising outcomes (death and serious injury, eg, or evidence of their own culpability and fallibility).^80^ Though some argue that poor outcomes during simulation may promote learning,^82^ the potential for psychological harm is there, not just for the intended targets of the improvement but also for other simulation stakeholders such as standardised patient actors or individuals managing the simulation.^84^ One argument is that these risks are acceptable if the right precautions are taken (a safe environment, both physically and psychologically, a good debriefing, avoiding death scenarios with early learners, providing follow-up after simulation or even mentioning the possibility of patient death during pre-briefing).^81–83^ Others argue for careful reflection on the objectives of the simulation and participants’ profiles.^80^

## Conclusions

This paper provides an overview of how simulation can be used in research aimed at studying how to improve quality and safety in healthcare. We propose that simulation has considerable potential; examples thus far show that it is capable of accommodating multiple research designs and allowing study of some situations that would otherwise escape scientific evaluation, such as rare events or situations where direct observation or experiments are not possible for ethical or other reasons. Simulation has the advantage of providing researchers with a controlled environment to test their hypotheses, and to do so safely for patients and participants. It allows for controlled variation of variables, a foundational element of empiric scientific knowledge.^45^

Given these strengths, it is no surprise that clinical simulation networks are including simulation-based research in their programmes of work beside simulation-based education and training,^4 5^ and that the use of simulation to improve quality and safety in healthcare is also gaining increased attention on the agenda of the simulation community.^85 86^ In this context, research *through* simulation^4^ opens promising perspectives for healthcare improvement research (box 6).

Box 6 **Recommendations**

- Simulation is a flexible and pluripotent technique that can be used in multiple study designs in healthcare improvement research.
- Researchers should consider simulation-based research when it is otherwise difficult, costly or ethically impossible to obtain data about a phenomenon.
- Researchers should evaluate the possibility of integrating simulation as part of a mixed-methods design, where the data obtained in the controlled environment of the simulation can be triangulated with empirical field data.
- Simulation-based research raises distinctive issues relating to study design, methods, and ethics that require ongoing attention.

Despite all its merits, SBR is not without its disadvantages. Its very artificiality affects the conclusions that can be drawn from the results of simulation studies, for example, in limiting what can be learnt about context. Overall, the influence of the simulation environment on the performance of participants is still relatively unknown,^9^ so caution is needed in drawing more general conclusions from simulation-based studies. Several authors highlight the need to match simulation design with simulation objectives,^16 19 69^ which supports the nuanced and reflexive approach to applicability/generalisability expressed in other disciplines.^63^ Until more is known, it seems sensible to generalise (with caution) the existence and direction of a relationship between variables established during simulation, but not the strength of this relationship.^44^

The review offered here sought to deepen understanding of a complex emerging area of research practice, and did not seek to be systematic. Transparency on the selection of articles^87^ is therefore limited. Future studies could complement our approach with a more systematic review of SBR in healthcare improvement research. This would help to further assess the current state of simulation-based improvement research.

In conclusion, simulation has the potential to become a useful addition to improvement researchers’ methodological toolkits. The main value simulation can bring to improvement research is by helping collect data on phenomena that researchers can hardly observe. In this way, simulation can help describe individual and organisational behaviour, generate theory and evaluate improvement interventions.
